# Treatment to reduce vascular calcification in hemodialysis patients using vitamin K (Trevasc-HDK): A study protocol for a randomized controlled trial

**DOI:** 10.1097/MD.0000000000021906

**Published:** 2020-09-04

**Authors:** Sabrina-Wong-Peixin Haroon, Bee-Choo Tai, Lieng-Hsi Ling, Lynette Teo, Andrew Davenport, Leon Schurgers, Boon-Wee Teo, Priyanka Khatri, Ching-Ching Ong, Sanmay Low, Xi-Er Yeo, Jia-Neng Tan, Srinivas Subramanian, Horng-Ruey Chua, Swee-Yaw Tan, Weng-Kin Wong, Titus-Wai-Leong Lau

**Affiliations:** a Division of Nephrology, National University Hospital Singapore; b Saw Swee Hock School of Public Health; c National University Heart Center; d Department of Diagnostic Imaging, National University Hospital, Singapore; e UCL Centre for Nephrology, Royal Free Hospital, University College London, United Kingdom; f Department of Biochemistry, Cardiovascular Research Institute Maastricht, The Netherlands; g Department of Medicine, Ng Teng Fong General Hospital; h Kidney and Medical Clinic Pte Ltd, Parkway East Hospital; i National Heart Center Singapore, Singapore.

**Keywords:** hemodialysis, vascular calcification, vitamin K2

## Abstract

**Introduction:**

End stage renal failure patients on hemodialysis have significant vascular calcification This is postulated to be related to sub-clinical vitamin K deficiency, which is prevalent in hemodialysis patients. Vitamin K deficiency result in the failure of the matrix GLA protein (MGP) to undergo carboxylation. MGP is a natural local inhibitor of vascular calcification and the lack of functional carboxylated MGP may contribute to increase vascular calcification. Vitamin K supplement should therefore correct this anomaly and decrease the rate or severity of vascular calcification in this population of patients on long-term maintenance hemodialysis. Our study seeks to evaluate the prevalence and the progression of vascular calcification in a cohort of maintenance hemodialysis patients. It will also evaluate the efficacy of vitamin K supplementation in reducing the progression of vascular calcification in this group of patients.

**Methods:**

This will be a single-center randomized, prospective and open-label interventional clinical trial of end stage renal failure patients on hemodialysis. We aim to recruit 200 patients. Eligible patients will be randomized to either the standard care arm or active treatment arm. Active treatment arm patients will receive standard care plus supplementation with oral vitamin K2 isoform 360 mcg 3 times weekly for a total duration of 18 months. Primary outcome measured will be absolute difference in coronary artery calcification score at 18-month between control and intervention arms. Secondary outcomes will be to compare absolute difference in aortic valve calcification, percentage of patients with regression of coronary artery calcification of at least 10%, absolute difference in aortic and systemic arterial stiffness, mortality from any cause and major adverse cardiovascular over the same period.

**Discussion:**

Evidence of successful regression or retardation of vascular calcification will support the conduct of larger and longer-term trials aimed at reducing cardiovascular disease mortality and major adverse cardiovascular events in this high-risk population using a safe and inexpensive strategy

**Trial registration:**

ClinicalTrials.gov NCT02870829. Registered on 17 August 2016 – Retrospectively registered, https://clinicaltrials.gov/ct2/show/NCT02870829

National University Hospital's Institutional Review Board (2015/01000)

## 1 Introduction

It is well recognized that coronary artery calcification (CAC), a form of vascular calcification (VC), occurs at a much higher rate in the dialysis population compared to age-matched populations without chronic kidney disease (CKD).^[1,2]^ Studies have also confirmed a positive association of CAC with adverse outcomes in this population.^[2]^ There is a dose relationship reported between the CAC score and cardiovascular disease (CVD) mortality; the higher the score, the higher the mortality risk.^[3]^ Given that one major cause of death and hospitalization in the dialysis population is CVD, these observations may be of critical importance in efforts to reduce CVD mortality and morbidity.^[2]^

Two distinct types of VC are reported in the CKD population.^[4]^ Atherosclerotic calcification occurs in the intimal layer and is associated with atherosclerosis and potentially rupture of unstable plaque, leading to acute myocardium infarction and stroke. Various animal models and human studies support the development of this form of VC in uremic and non-uremic settings. Another form, medial calcification (calcific arteriosclerosis) is also commonly observed in CKD and uremia, and contributes to a higher CVD mortality in this population due to increased vascular stiffness with loss of vessel elasticity, resulting in increased cardiac afterload, progressive left ventricular hypertrophy and ultimately, heart failure and arrhythmia.^[5]^

In the general non-CKD population, established risk factors for CAC are older age, diabetes, hypertension, dyslipidemia and smoking, while in CKD, dialysis vintage is an additional risk factor.^[6]^ Several factors unique to the uremic population appear to increase the risk of medial calcification which differs in pathogenesis from intimal atherosclerotic calcification, being typically non-inflammatory in nature.^[7]^ Hyperphosphatemia, elevated calcium-phosphate product and use of calcium-containing phosphate binders (a reflection of excess calcium loading) have been associated with an increased risk of VC in the CKD population. 1,25 (OH)2 Vitamin D3 supplementation, commonly employed to suppress parathyroid hormone in CKD, has also been implicated as a cause of VC.^[8]^ Other promoters (such as bone morphogenetic protein and osteocalcin) and inhibitors (such as matrix Gla protein and fetuin-A) of VC have been identified.^[9–12]^ While their role in clinical management remains to be established, these proteins are potential therapeutic targets in CKD-associated VC.

Given that CVD is a major cause of mortality and morbidity in the CKD population, there has been intense interest in therapies to reduce CVD events. Statin therapy has proven very successful in reducing major adverse cardiac events (MACE). However, two large prospective randomized controlled trials could not demonstrate a significant reduction in CVD, non-fatal myocardial infarction and non-fatal stroke in patients on hemodialysis assigned to potent hypolipidemic agents (namely atorvastatin and rosuvastatin) despite significant LDL-cholesterol lowering compared to placebo.^[13,14]^ Control of other CVD risk factors which can accelerate VC is routine in the management of patients on dialysis. However, there are no randomized trials investigating the effect of glycemic or blood pressure control on VC or CAC in this population.

The latest frontier in the battle against VC and CVD in the dialysis population is CKD-mineral bone disorder which is characterized by excessive calcium loading, phosphate retention and hyperparathyroidism.^[15]^ In vitro as well as population studies support the role of each of these factors, alone or in combination, in the genesis of VC.^[16,17]^ Therefore, efforts to reduce VC in the CKD population have focused on correcting the altered bone mineral homeostasis. Reduction in calcium load is achieved by using lower dialysate calcium concentrations and non-calcium containing phosphate binders such as sevelamer.^[18,19]^ Lowering of serum phosphate in CKD patients with elevated phosphate level is also key to reducing the onset and severity of VC. There has been some success with the use of calcimimetics in reversing VC in dialysis patients.^[20]^ However, the largest randomized controlled trial to date did not show a statistically significant reduction in CVD outcomes in hemodialysis patients with moderate to severe hyperparathyroidism receiving cinacalcet over a median of 21 months compared to placebo.^[21]^

The cellular and molecular events leading to calcium deposition in vascular tissue are highly regulated. Animal and molecular studies have demonstrated that vitamin K can influence the development and progression of VC, via its involvement in the carboxylation of matrix GLA protein (MGP).^[22,23]^ MGP, a protein synthesized by vascular smooth muscle cells, functions as a natural local inhibitor of VC^[24,25]^ but becomes functional only after vitamin K2-dependent carboxylation. MGP deficient mice have been shown to develop massive calcification and do not survive beyond 6 weeks of birth.^[26]^ Many dialysis patients may have sub-clinical vitamin K deficiency, possibly related to dietary restriction, as evidenced by the high level of dephosphorylated undercarboxylated MGP (dp-ucMGP) measured in this population.^[27]^

Oral vitamin K supplementation can enhance carboxylation of MGP and lower plasma dp-ucMGP. Vitamin K1 (phylloquinone) is the usual dietary source of vitamin K. Vitamin K2 is derived from enterocyte conversion of vitamin K1 or from indigenous gut bacteria production^[28]^ although 1 food source, natto (soybean fermented using Bacillus subtilis) has a very high content of vitamin K2.^[29]^ The precise dose of vitamin K needed in the physiological state to activate all vitamin K dependent proteins is not entirely clear. There is also an inherent divergent use of vitamin K1 for carboxylation of vitamin K dependent proteins in the liver such as for coagulation factors, preferentially over its utilization in the periphery (extra-hepatic), as a mean of ensuring adequacy of certain essential protein function for short-term survival.^[30]^ This preferential triaging of the utilization of vitamin K may result in significant reduction of vitamin-K dependent carboxylation of extra-hepatic vitamin-K proteins even when modest deficiency exists. Peripheral carboxylation of vitamin K dependent proteins such as MGP are dependent on long-chain isoforms of vitamin K2 (menaquinones). One such isoform with proven efficacy in decreasing dp-ucMGP levels is menaquinone-7 (MK-7).^[27]^

A recent randomized trial reporting 1-year outcome of vitamin K2 supplementation in hemodialysis patients found no effect on aortic calcification despite reduction in dp-ucMGP.^[31]^ There are still two ongoing trials evaluating the impact of vitamin K1 supplementation on VC in end stage renal failure (ESRF) and 1 with vitamin K2 supplementation in ESRF patients with non- valvular atrial fibrillation.^[32–34]^ All these trials were conducted in the Caucasian populations. As vitamin K intake will vary according to diet, there may well be differences between European and North American patient groups compared to those from Asia. To our knowledge, there are no similar studies in Asians. The proposed “Treatment to Reduce Vascular Calcification in Hemodialysis Patients Using Vitamin K” (Trevasc-HDK) study is therefore timely.

## 2 Objectives

The primary objective of Trevasc-HDK is to evaluate the effect of vitamin K2 (MK-7) supplementation on the progression of CAC in ESRF patients on HD. We hypothesize that oral MK-7 will enhance carboxylation of MGP, lower levels of dp-ucMGP and reduce CAC and/or aortic vascular calcification (AVC) in the HD population. Secondary outcomes of interest include the effects of MK-7 on AVC (known to be correlated with CAC), aortic stiffness (which is determined by aortic calcification rather than atherosclerotic plaque burden) and clinical outcomes (see “Study outcomes” below). Trevasc-HDK will also provide the first local data on the prevalence and severity of CAC or AVC in patients on maintenance HD, as well as the natural history of calcification in those not receiving MK-7.

## 3 Methods

**Study design** Trevasc-HDK is a single-center randomized, prospective and open-label interventional clinical trial of ESRF patients on HD. The study protocol was developed in adherence to the Good Clinical Practice Guidelines and Standard Protocol Items: Recommendations for Intervention Trials.^[35]^ The study is performed according to the ethical standards of the National University Hospital's Institutional Review Board (2015/01000) on clinical trials with human subjects and with the Helsinki Declaration of 1975 (and as revised in1983). The trial is registered at ClinicalTrials.gov (NCT02870829).

### 3.1 Study setting:

National University Hospital is 1 of the major academic tertiary hospitals in Singapore caring for ESRF patients. Potential study subjects are identified from the Haemodialysis and Nephrology clinics in this hospital (single site). All patients will continue to receive standard of care dialysis at their usual satellite dialysis units during the conduct of this study.

### 3.2 Eligibility criteria:

Patients meeting the inclusion and exclusion criteria will be invited to participate and written informed consent obtained for those willing to participate. Consented subjects will attend a screening visit (V0) where a multislice computed tomogram will be performed to assess their degree of CAC. As the main objective is to investigate the effects of intervention on VC, pre-existing CAC indicated by a baseline CAC score of ≥ 30 Agatston units is mandated for entry into the trial. This threshold also excludes patients with mild CAC which is unlikely to progress in the relatively short window of observation.^[19]^ Other inclusion and exclusion criteria are outlined in Table 1. Consent is taken by study team co- investigator after detailed explanation of purpose, benefit and risk of the study. Witness is required for patient signing informed consent in Chinese and Malay language. Patients are informed the right to withdraw consent and participation at any time during the study period.

**Table 1:**
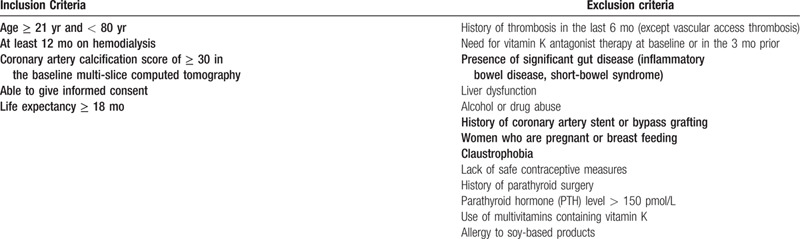
Inclusion and exclusion criteria.

### 3.3 Study interventions

Eligible patients will be randomized to either the control arm (standard care) or active treatment arm (standard care plus supplementation with vitamin K2 isoform MK-7). The dose of MK-7 chosen is oral 360 mcg 3 times weekly for a total duration of 18 months.

Patients will be randomly allocated into control and intervention arms in a 1:1 fashion using random permuted blocks of size 4 and 6. The randomization will be stratified by diabetes status. The sequence of treatment allocation will be generated using the *ralloc* module of the STATA software. Randomization will be performed by clinical trial unit coordinator during office hour.

### 3.4 Sample size

To ensure that the study is adequately powered, we have estimated the sample size based on the primary outcome of absolute difference in CAC score at 18-months between the control and intervention arms, assuming mean CAC scores at 18-months of 1005 and 786 respectively as reported by Block et al.^[19]^ Applying natural logarithmic transformation to CAC scores due to skewed distribution, this corresponds to a mean logCAC of 6.91 and 6.67, respectively. Further assuming a common SD of 0.55, power of 80%, 2-sided level of significance of 5% and equal allocation between the 2 treatment arms, a minimum sample size of 170 in total, or 85 per arm will be required. Accounting for an attrition rate of 15%, we will recruit a total of 200 patients.

### 3.5 Study workflow

Randomized patients will be scheduled for a first visit (V1) within 4 weeks from V0, during which blood will be drawn for dp-ucMGP, the biomarker of vitamin K deficiency, and aortic stiffness measured. Patients who are randomized to the treatment arm will receive MK-7 during this visit.

During the treatment phase of 18 months, the primary physician will review the study subjects at intervals of 2-6 months as per standard care. The research coordinator will contact these subjects every month to motivate compliance with active treatment, and perform pill counts during clinic visits to verify adherence. The investigational product (vitamin K2 isoform MK-7) will be stored in accordance to manufacturer's recommendations at Investigational Medicine Unit, National University Hospital Singapore.

**Figure 1:**
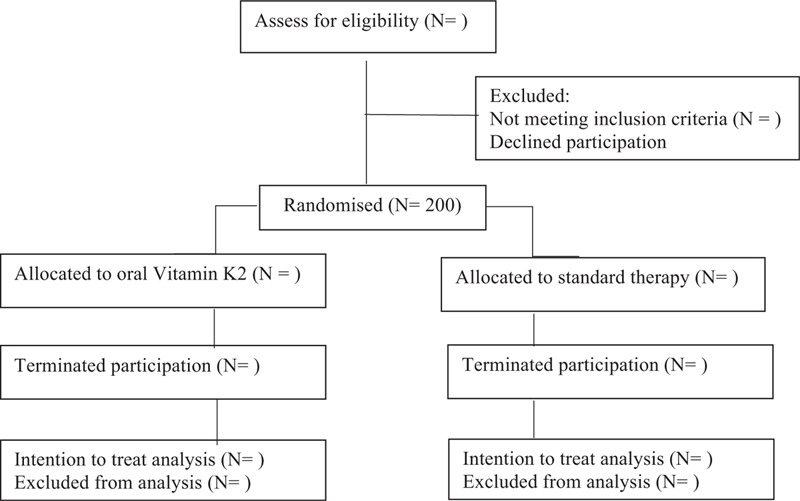
Flow chart of the trial.

**Figure 2:**
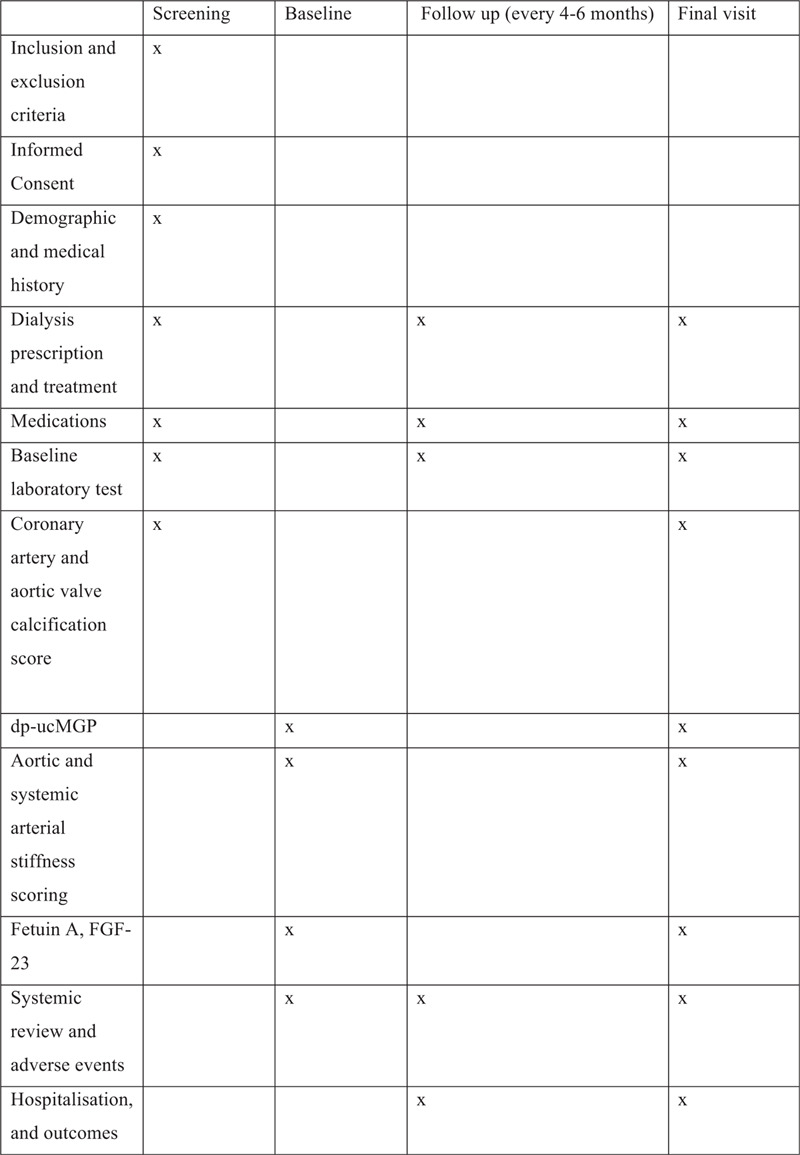
Study schedule of assessment.

Active treatment will end after 18 months, following which an end of study visit (V2) will be scheduled for all study subjects within 4 weeks. During V2, blood will be obtained for dp-ucMGP, and measurement of aortic stiffness and CAC/AVC scores repeated. The trial flow chart and study schedule is summarized in Figures 1 and 2 respectively.

The criteria for termination of the study are:

(1) Withdraw consent
(2) completion of the study protocol at 18 months
(3) renal transplantation
(4) mortality
(5) requirement for Vitamin K antagonist
(6) allergy to MK-7
(7) parathyroidectomy
(8) started on any vitamin K supplementation outside of clinical trial

### 3.6 Biomarker of sub-clinical vitamin K deficiency

Blood will be collected in an ethylenediaminetetraacetic acid tube, processed after withdrawal and stored at −80 °C as plasma before analysis at Coagulation Profile BV, Maastricht University, the Netherlands. In brief, plasma dp-ucMGP levels will be determined in a single run using the commercially available IVD CE-marked chemiluminescent InaKtif MGP assay on the IDS-iSYS system (IDS, Boldon). Both patient samples and internal calibrators will be incubated with magnetic particles coated with murine monoclonal antibodies against dp-MGP, acridinium-labelled murine monoclonal antibodies against ucMGP, and an assay buffer. The magnetic particles will be captured using a magnet and washed to remove any unbound analyte. Trigger reagents will be added; the resulting light emitted by the acridinium label is directly proportional to the level of dp-ucMGP in the sample^[36]^

### 3.7 Measurement of aortic stiffness

Conduit arterial or aortic stiffness will be assessed using applanation tonometry (SphygmoCorVx, AtCor Medical, West Ryde, NSW, Australia)^[37]^ by trained technicians blinded to treatment assignment. This non-invasive technique uses a high-fidelity pressure transducer applied lightly over the carotid, femoral and radial arteries to obtain the following:

#### 3.7.1 a. Carotid-femoral pulse wave velocity (cfPWV)

The right carotid to right femoral path length will be measured in a straight line with a tape ruler, and 80% of this distance used as pulse wave traveled distance (d).^[38]^ Right carotid-femoral transit time will be obtained by subtracting the time (t) between onset of the electrocardiographic R wave and the foot of the carotid pulse and the time between the R-wave and the femoral pulse, each averaged from 8 to 10 sequential waveforms. cfPWV will be calculated as d/t and expressed as m/s. Two measurements will be taken and averaged. If their difference exceeds 0.5 m/s, a third measurement will be made and the median value taken.

#### 3.7.2 b. Pulse wave analysis

This is performed on the arm not used for vascular access. From the radial artery waveform obtained by the high-fidelity tonometer, the SphygmoCorPx System reconstructs the aortic pressure waveform using a transfer function.^[39,40]^ This waveform depends on left ventricular ejection, as well as the timing and amount of wave reflection from branch points or areas of impedance mismatch which are determined by aortic stiffness and arteriolar tone.^[41]^ From the aortic waveform, central systolic, diastolic, mean and pulse pressures are obtained. Aortic augmentation index (AI) will be calculated as the increment in pressure from the first systolic shoulder of the ascending aortic pressure wave to the peak of the second, late systolic shoulder, expressed as a percentage of the pulse pressure. Aortic AI is also normalized to a heart rate of 75 beats/min to facilitate comparison.

#### 3.7.3 Multislice CT

Calcium scoring will be performed using a high-pitch multislice computed tomogram scanner. All patients should have a resting heart rate <100 beats/min. Scan length is defined by a scout view from the arch to the diaphragm. For all scans, the extent of CAC and AVC will be assessed using both volume and Agatston scores by 2 radiologists unaware of treatment assignment. The volume score will be determined by the calculated volume of calcified plaques with a density of > 130 Hounsfield units and a minimum size of 0.5 mm^3^ based on isotropic interpolation.^[42]^ The Agatston score will be calculated by multiplying the weighted value and size of the calcified areas as previously described.^[43]^

### 3.8 Study outcomes

Control and intervention arms will be compared for the following outcomes of interest:

Primary outcome

Absolute difference in CAC score at 18-months

Secondary outcomes

(1) Absolute difference in AVC score at 18-months
(2) Percentage of patients with regression of CAC of ≥ 10% over 18-month
(3) Absolute difference in cfPWV and AI at 18-months
(4) Mortality from any cause within the study period
(5) MACE defined as non-fatal myocardial infarction, heart failure, acute coronary syndrome, need for coronary revascularization, non-fatal stroke, significant peripheral vascular disease (gangrene, amputation, need for revascularization) within the study period
(6) Difference in plasma levels of dp-ucMGP at 18 months
(7) Vascular access events, including clotting requiring insertion of a new dialysis catheter, declotting or exchange of an existing catheter, and vascular intervention during the study period.

### 3.9 Statistical methods

The absolute difference in CAC and AVC scores at 18-months between control and intervention arms will be compared using the t-test following log transformation. Adjustment for baseline CAC and AVC scores respectively will be made via the analysis of covariance. Likewise, the effects of MK-7 supplementation on dp-ucMGP level, CFPWV and AI at 18 months will be compared between the 2 groups using the t-test, with adjustment made for the respective baseline covariates via analysis of covariance.

The percentage of patients with regression of CAC score of ≥ 10% at 18 months from baseline will be compared between control and intervention arms via the χ^2^ test. The effect of treatment will be quantified using the odds ratio estimate and its associated 95% confidence interval. The logistic regression analysis will be used to account for potential confounders where applicable.

For all-cause mortality and MACE, the time to the respective event is defined as the time from randomization to the time when the event of interest occurred. Subjects in whom an event was not observed will be censored at 18-months. The Kaplan-Meier survival analysis and the log-rank test will be used to compare the survival distributions between the control and intervention groups. The effect of treatment will be quantified using the hazard ratio estimate and its associated 95% confidence interval. Further, the Cox proportional hazards regression stratified by diabetes will be used to account for potential confounders where applicable.

All statistical evaluations will be made assuming a 2-sided test at the 5% level of significance based on an intention-to-treat analysis.

#### 3.9.1 Monitoring

Data will be reviewed monthly by study team to ensure accuracy and completeness. Both primary investigator and study team will perform data and safety monitoring. In addition, National University Hospital Research office team will perform data monitoring and quality checks at least twice a year. The primary investigator will be required to submit trial report to Health Science Authority regarding the progress of the trial and onsite monitoring visit are performed if necessary.

#### 3.9.2 Adverse event reporting and harm

Timely, accurate and complete reporting and analysis of safety information from clinical studies are crucial for the protection of patients and are mandated by regulatory agencies. Unanticipated Problems Involving Risks to Subjects or Others are events that include any incident, experience, or outcome (including adverse events) that are unexpected, related or possibly related to research participation, with risk of harm and are part of the safety reporting structure as mandated by our Instuitional Review Board. Data collected on adverse events will be evaluated thoroughly and if necessary, may lead to changes in the conduct of the study or even termination or suspension of the study.

Due to the nature of the study being open-label in design, the Unanticipated Problems Involving Risks to Subjects or Others events and adverse events will be reported formally only for patients on the active treatment group. Those randomized to the control group will have all adverse events recorded (but not reported) for the purpose of comparison with the active treatment group.

### 3.10 Patient and public involvement

Patients and the public were not involved in the development of this study protocol. The conception of the study and the development of the protocol were all investigator initiated and driven. Results will be disseminated to all study participants after study completion. Results will also be made known to the wider medical community through peer-reviewed publications and presentations at national and international meetings.

## 4 Discussion

Majority (80%) of our local ESRF patients are on hemodialysis.^[44]^ Similar to many developed countries, cardiovascular diseases have now replaced infection as the major cause of mortality and mortality among end stage renal failure patients.^[45]^ The difference in pathophysiology of cardiovascular disease in renal failure patients compared to general population have raised questions about possible interventions or management. Kidney Diseases Improving Global Outcomes has recommended routine screening of vascular calcification for dialysis patients, but the subsequent plan of management remains unclear and controversial.^[46]^ Should we still adopt the general measures to reduce cardiovascular risk and refer to cardiology for intervention? Actual intervention with coronary stenting and angioplasty in addition to being invasive may not be as useful compared to the general population. As such we felt that it was important to study valvular calcification and vascular stiffness as this correlate closely with coronary artery calcification and has been reported to have an impact on morbidity and mortality for our patient.^[47,48]^

Recent evidence emphasizes the possibility of Vitamin K playing a key role in vascular calcification. Not surprising, end stage renal failure patients are known to be deficient in various nutrients due to dietary restrictions and losses with dialysis. We have designed the Trevasc- HDK trial to investigate the importance of Vitamin K on the natural history of CV disease, and whether intervention with Vitamin K2 can modulate progression.

Vitamin K preparations, predominantly Vitamin K1, are currently available as supplements over the counter in health food shops and pharmacies, but currently not prescribed to patient as medication. In our study Vitamin K2 is prescribed orally and to be administered 3x/week. As this is a clinical trial to study the role of Vitamin K, it was registered with our local Health Science Authority for reporting and monitoring purposes. As Vitamin K2 has greater biological activity, and conversion of Vitamin K1 to K2 can vary between individuals, we chose to administer Vitamin K2.

The advantages of our study, in addition to the primary outcome, we will be able to provide additional information about the possible Vitamin K deficiency in our patients’ diet. A previous short-term study demonstrated that oral administration of Vitamin K2 led to a change in dp-ucMGP activity, in a dose response manner.^[49]^ We chose the lowest dose of Vitamin K2 (360 mcg 3x/week) used in this dose finding study which had an effect on dp-ucMGP activity, as we have no current data about Vitamin K intake in our population and our Asian patients have a lower body surface area compared to Europeans, and no data on taking Vitamin K2 for 18 months. Although patients enrolled into the study are managed primarily by their own primary nephrologist and there should be minimal variation of management in both groups, as clinicians adhere to agreed management protocols.

Our trial is important even if the study is negative as we will have more insight into the baseline level and role of vitamin Kin Asian patients as well as provide us with the magnitude and progression of calcification in our multiethnic HD population.

Trevasc-HDK will be the first randomized, controlled trial to test the hypothesis that oral vitamin K2 supplementation can reduce VC in an Asian HD population. Evidence of successful regression or retardation of VC will support the conduct of larger and longer-term trials aimed at reducing CVD mortality and MACE in this high-risk population using a safe and inexpensive strategy.

## 5 Study status

The latest protocol version is 2.0. The revised protocol includes changes in the designated laboratory to process the biomarkers, additional tests done and change in inclusion criteria to include patients with lower dialysis vintage of 12 months. Our study started the first patient recruitment on August 10, 2016. As we did not reach recruitment target within the initial specified time period, we extended the recruitment period and last patient recruited was on July 6, 2017. All patients have completed their final visit and we are currently pending biomarker analysis. Due to current COVID-19 pandemic, we anticipate a slight delay in trial closure. The current projection is that the trial should be completed by late 2020.

## Acknowledgments

The Trevasc-HDK research team would like to thank Nattopharma ASA for providing Vitamin K2 for the trial. We are grateful to the National University Health System's Investigational Medicine Unit, Department of Diagnostic Imaging and Cardiovascular Imaging Core Laboratory for their role in the execution of this trial.

## Author contribution

All authors have contributed to the development of the protocol and study design as members of the research team. The article was draft by SH, TBC, LLH, LTLS and TL. The manuscript was revised by AD, LS, PK, OCC, LS, YEX, TJN, SS, CHR, TSY, WWK and TBW. All the authors approved the final version of the manuscript and are accountable for the author's own contributions, accuracy or integrity of the manuscript.

**Conceptualization:** Sabrina Haroon, Titus Lau.

**Funding acquisition:** Sabrina Haroon.

**Investigation:** Sabrina Haroon, Titus Lau.

**Methodology:** Sabrina Haroon, Bee Choo Tai, Lieng Hsi Ling, Lynette Teo, Ching Ching Ong, Titus Lau.

**Project administration:** Sabrina Haroon.

**Supervision:** Bee Choo Tai, Lieng Hsi Ling, Lynette Teo, Boon Wee Teo.

**Visualization:** Titus Lau.

**Writing – original draft:** Sabrina Haroon, Titus Lau.

**Writing – review & editing:** Sabrina Haroon, Bee Choo Tai, Lieng Hsi Ling, Lynette Teo, Leon Schurgers, Boon Wee Teo, Priyanka Khatri, Ching Ching Ong, Sanmay Low, Xi Er Yeo, Jia Neng Tan, Srinivas Subramanian, Horng Ruey Chua, Swee Yaw Tan, Weng Kin Wong, Titus Lau.

## References

[1] Goodman WG Goldin J Kuizon BD Coronary-artery calcification in young adults with end-stage renal disease who are undergoing dialysis. N Engl J Med 2000;342:1478 83. 10816185 10.1056/NEJM200005183422003

[2] Raggi P Boulay A Chasan-Taber S Cardiac calcification in adult hemodialysis patients. A link between end-stage renal disease and cardiovascular disease? J Am Coll Cardiol 2002;39:695 701. 11849871 10.1016/s0735-1097(01)01781-8

[3] Matsuoka M Iseki K Tamashiro M Impact of high coronary artery calcification score (CACS) on survival in patients on chronic hemodialysis. Clin Exp Nephrol 2004;8:54 8. 15067517 10.1007/s10157-003-0260-0

[4] Mizobuchi M Dwight Towler D Slatopolsky E Vascular calcification: the killer of patients with chronic kidney disease. J Am Soc of Nephrol 2009;20:1453 64. 19478096 10.1681/ASN.2008070692

[5] Shroff R Long DA Shanahan C Mechanistic insights into vascular calcification in chronic kidney disease. J Am Soc of Nephrol 2013;24:179 89. 23138485 10.1681/ASN.2011121191

[6] London GM Marchais SJ Guérin AP Arteriosclerosis, vascular calcifications and cardiovascular disease in uremia. Curr Opin Nephrol Hypertens 2005;14:525 31. 16205470 10.1097/01.mnh.0000168336.67499.c0

[7] Schlieper G Schurgers L Brandenburg V Vascular calcification in chronic kidney disease: an update. Nephrol Dial Transplant 2016;31:31 9. 25916871 10.1093/ndt/gfv111

[8] Nigwekar SU Thadhani R Brandenburg VM Calciphylaxis. N Eng J Med 2018;378:1704 14. 10.1056/NEJMra1505292 29719190

[9] Shroff RC Shah V Hiorns MP The circulating calcification inhibitors, fetuin-A and osteoprotegerin, but not matrix Gla protein, are associated with vascular stiffness and calcification in children on dialysis. Nephrol Dial Transplant 2008;23:3263 71. 18463323 10.1093/ndt/gfn226

[10] Viegas CS Santos L Macedo AL Chronic kidney disease circulating calciprotein particles and extracellular vesicles promote vascular calcification: a role for GRP (Gla-Rich Protein). Arterioscler Thromb Vasc Biol 2018;38:575 87. 29301790 10.1161/ATVBAHA.117.310578

[11] Evrard S Delanaye P Kamel S Vascular calcification: from pathophysiology to biomarkers. Clinica Chimica Acta 2015;438:401 14. 10.1016/j.cca.2014.08.034 25236333

[12] Malhotra R Burke MF Martyn T Inhibition of bone morphogenetic protein signal transduction prevents the medial vascular calcification associated with matrix Gla protein deficiency. PLoS One 2015;10:e0117098. 25603410 10.1371/journal.pone.0117098 PMC4300181

[13] Baigent C Landray MJ Reith C The effects of lowering LDL cholesterol with simvastatin plus ezetimibe in patients with chronic kidney disease (Study of Heart and Renal Protection): a randomized placebo-controlled trial. Lancet 2011;377:2181 92. 21663949 10.1016/S0140-6736(11)60739-3 PMC3145073

[14] Wanner C Krane V März W (German Diabetes and Dialysis Study Investigators). Atorvastatin in patients with type 2 diabetes mellitus undergoing hemodialysis. N Engl J Med 2005;353:238 48. 16034009 10.1056/NEJMoa043545

[15] Moe SM Chen NX Mechanisms of vascular calcification in chronic kidney disease. J Am Soc Nephrol 2008;19:213 6. 18094365 10.1681/ASN.2007080854

[16] Reynolds JL Joannides AJ Skepper JN Human vascular smooth muscle cells undergo vesicle-mediated calcification in response to changes in extracellular calcium and phosphate concentrations: a potential mechanism for accelerated vascular calcification in ESRD. J Am Soc Nephrol 2004;15:2857 67. 15504939 10.1097/01.ASN.0000141960.01035.28

[17] Ketteler M Fetuin-A and extraosseous calcification in uremia. Curr Opin Nephrol Hypertens 2005;14:337 42. 15931001 10.1097/01.mnh.0000172719.26606.6f

[18] Chertow GM Burke SK Raggi P Sevelamer attenuates the progression of coronary and aortic calcification in hemodialysis patients. Kidney Int 2002;62:245 52. 12081584 10.1046/j.1523-1755.2002.00434.x

[19] Block GA Spiegel DM Ehrlich J Effects of sevelamer and calcium on coronary artery calcification in patients new to hemodialysis. Kidney Int 2005;68:1815 24. 16164659 10.1111/j.1523-1755.2005.00600.x

[20] Raggi P Chertow G Torres PU The ADVANCE study: a randomized study to evaluate the effects of cinacalcet plus low-dose vitamin D on vascular calcification in patients on hemodialysis. Nephrol Dial Transplant 2011;26:1327 39. 21148030 10.1093/ndt/gfq725

[21] EVOLVE Trial Investigators Effect of cinacalcet on cardiovascular disease in patients undergoing dialysis. N Engl J Med 2012;367:2482 94. 23121374 10.1056/NEJMoa1205624

[22] Proudfoot D Shanahan CM Molecular mechanisms mediating vascular calcification: role of matrix Gla protein. Nephrology 2006;11:455 61. 17014561 10.1111/j.1440-1797.2006.00660.x

[23] Schurgers LJ Cranenburg EC Vermeer C Matrix Gla-protein: the calcification inhibitor in need of vitamin K. Thromb Haemost 2008;100:593 603. 18841280

[24] Theuwissen E Smit E Vermeer C The role of vitamin K in soft-tissue calcification. Adv Nutr 2012;3:166 73. 22516724 10.3945/an.111.001628 PMC3648717

[25] Murshed M Schinke T McKee MD Extracellular matrix mineralization is regulated locally; different roles of two gla-containing proteins. J Cell Biol 2004;165:625 30. 15184399 10.1083/jcb.200402046 PMC2172384

[26] Luo G Ducy P McKee MD Spontaneous calcification of arteries and cartilage in mice lacking matrix GLA protein. Nature 1997;386:78 81. 9052783 10.1038/386078a0

[27] Westenfeld R Krueger T Schlieper G Effect of vitamin K2 supplementation on functional vitamin k deficiency in hemodialysis patients: a randomized trial. Am J Kidney Dis 2012;59:186 95. 22169620 10.1053/j.ajkd.2011.10.041

[28] Shearer MJ Newman P Metabolism and cell biology of vitamin K. Thromb Haemost 2008;100:530 47. 18841274

[29] Sumi H Accumulation of vitamin K2 (menaquinone-7) in plasma after ingestion of Natto and Natto Bacilli (B. Subtilis natto). Food Sci Technol Res 1999;5:48 50.

[30] McCann JC Ames BN Vitamin K an example of triage theory: is micronutrient inadequacy linked to diseases of aging? Am J Clin Nutr 2009;90:889 907. 19692494 10.3945/ajcn.2009.27930

[31] Oikonomaki T Papasotiriou M Ntrinias T The effect of vitamin K2 supplementation on vascular calcification in haemodialysis patients: a 1-year follow-up randomized trial. Int Urol Nephrol 2019;51:2037 44. 31529295 10.1007/s11255-019-02275-2

[32] Krueger T Schlieper G Schurgers L Vitamin K1 to slow vascular calcification in hemodialysis patients (VitaVasK trial): a rationale and study protocol. Nephrol Dial Transplant 2014;29:1633 8. 24285427 10.1093/ndt/gft459

[33] Holden RM Booth SL Day AG Inhibiting the progression of arterial calcification with vitamin K in HemoDialysis patients (iPACK-HD) trial: rationale and study design for a randomized trial of vitamin K in patients with end stage kidney disease. Can J Kidney Health Dis 2015;2:53. 26075081 10.1186/s40697-015-0053-x PMC4465015

[34] De Vriese AS Caluwé R Pyfferoen L Multicenter randomized controlled trial of vitamin K antagonist replacement by rivaroxaban with or without vitamin K2 in hemodialysis patients with atrial fibrillation: the Valkyrie Study. J Am Soc Nephrol 2020;31:186 96. 31704740 10.1681/ASN.2019060579 PMC6935010

[35] Chan A-W Tetzlaff JM Gøtzsche PC SPIRIT 2013 explanation and elaboration: guidance for protocols of clinical trials. BMJ 2013;346:e7586. 23303884 10.1136/bmj.e7586 PMC3541470

[36] Delanaye P Krzesinski JM Warling X Dephosphorylated-uncarboxylated Matrix Gla protein concentration is predictive of vitamin K status and is correlated with vascular calcification in a cohort of hemodialysis patients. BMC Nephrol 2014 Dec 1;15:145. 25190488 10.1186/1471-2369-15-145 PMC4174604

[37] Chirinos JA Arterial stiffness: basic concepts and measurement techniques. J Cardiovasc Transl Res 2012;5:243 55. 22447229 10.1007/s12265-012-9359-6

[38] Van Bortel LM Laurent S Boutouyrie P Expert consensus document on the measurement of aortic stiffness in daily practice using carotid-femoral pulse wave velocity. J Hypertens 2012;30:445 8. 22278144 10.1097/HJH.0b013e32834fa8b0

[39] Chen CH Nevo E Fetics B Estimation of central aortic pressure waveform by mathematical transformation of radial tonometry pressure: Validation of generalized transfer function. Circulation 1997;95:1827 36. 9107170 10.1161/01.cir.95.7.1827

[40] Pauca AL O’Rourke MF Kon ND Prospective evaluation of a method for estimating ascending aortic pressure from the radial artery pressure waveform. Hypertension 2001;38:932 7. 11641312 10.1161/hy1001.096106

[41] Murgo JP Westerhof N Giolma JP Aortic input impedance in normal man: relationship to pressure wave forms. Circulation 1980;62:105 16. 7379273 10.1161/01.cir.62.1.105

[42] Moe SM O’Neill KD Fineberg N Assessment of vascular calcification in ESRD patients using spiral CT. Nephrol Dial Transplant 2003;18:1152 8. 12748349 10.1093/ndt/gfg093

[43] Agatston AS Janowitz WR Hildner FJ Quantification of coronary artery calcium using ultrafast computed tomography. J Am Coll Cardiol 1990;15:827 32. 2407762 10.1016/0735-1097(90)90282-t

[44] Singapore renal registry annual report 2015. National Disease Registry Office. Available at: https://www.nrdo.gov.sg/docs/librariesprovider3/default-document-library/singapore-renal-registry-annual-report-2016_1999-till-2016_v5_online_final.pdf?sfvrsn=0. Accessed May 1, 2016.

[45] United States Renal Data System 2015. Available at: https://www.usrds.org/2015/download/vol1_03_Morbidity_Mortality_15.pdf. Accessed May 1, 2016.

[46] Isakova T Nickolas TL Denburg M KDOQI US commentary on the 2017 KDIGO clinical practice guideline update for the diagnosis, evaluation, prevention, and treatment of chronic kidney disease–mineral and bone disorder (CKD-MBD). Am J Kidney Dis 2017;70:737 51. 28941764 10.1053/j.ajkd.2017.07.019

[47] Koulaouzidis G Nicoll R MacArthur T Coronary artery calcification correlates with the presence and severity of valve calcification. Int J Cardiol 2013;168:5263 6. 23993324 10.1016/j.ijcard.2013.08.019

[48] Guo J Fujiyoshi A Willcox B Increased aortic calcification is associated with arterial stiffness progression in multiethnic middle-aged men. Hypertension 2017;69:102 8. 27821619 10.1161/HYPERTENSIONAHA.116.08459 PMC5145727

[49] Caluwé R Vandecasteele S Van Vlem B Vitamin K2 supplementation in haemodialysis patients: a randomized dose-finding study. Nephrology Dialysis Transplantation 2014;29:1385 90. 10.1093/ndt/gft464 24285428

